# Novel Virus Related to Kaposi’s Sarcoma–Associated Herpesvirus from Colobus Monkey

**DOI:** 10.3201/eid2508.181802

**Published:** 2019-08

**Authors:** Akshay Dhingra, Tina Ganzenmueller, Elias Hage, Nicolás M. Suárez, Kerstin Mätz-Rensing, Dimitri Widmer, Stefan Pöhlmann, Andrew J. Davison, Thomas F. Schulz, Artur Kaul

**Affiliations:** Hannover Medical School Institute of Virology, Hannover, Germany (A. Dhingra, T. Ganzenmueller, E. Hage, T.F. Schulz);; German Centre for Infection Research, Hannover-Braunschweig Site, Germany (A. Dhingra, T. Ganzenmueller, E. Hage, T.F. Schulz);; University Hospital Tübingen, Tübingen, Germany (T. Ganzenmueller);; MRC–University of Glasgow Centre for Virus Research, Glasgow, Scotland, UK (N.M. Suárez, A.J. Davison);; German Primate Center–Leibniz Institute for Primate Research, Göttingen, Germany (K. Mätz-Rensing, S. Pöhlmann, A. Kaul);; Zoo Dresden GmbH, Dresden, Germany (D. Widmer);; University Göttingen, Göttingen (S. Pöhlmann)

**Keywords:** Virology, bioinformatics, evolution, viruses, Kaposi sarcoma, herpesvirus, colobine gammaherpesvirus 1, macaque, monkey, Kaposi’s sarcoma–associated herpesvirus, Germany

## Abstract

We determined the complete genome sequence of a virus isolated from a mantled guereza that died of primary effusion lymphoma. The virus is closely related to Kaposi’s sarcoma–associated herpesvirus (KSHV) but lacks some genes implicated in KSHV pathogenesis. This finding may help determine how KSHV causes primary effusion lymphoma in humans.

Kaposi’s sarcoma–associated herpesvirus (KSHV) causes Kaposi sarcoma, primary effusion lymphoma, and the plasma cell variant of multicentric Castleman disease in humans (*1*). KSHV-related viruses (also known as rhadinoviruses) naturally infect New and Old World primates (*2*–*5*). Old World primate rhadinoviruses fall into 2 lineages, rhadinovirus 1 (RV1) and rhadinovirus 2 (RV2) (*2*,*6*). The RV1 lineage contains KSHV; the retroperitoneal fibromatosis–associated herpesviruses (RFHVs) identified in *Macaca nemestrina*, *M. fascicularis*, and *M. mulatta* macaques; and closely related viruses of other Old World primates (*2*,*5*). The RV2 lineage contains macaque viruses more distantly related to KSHV, such as rhesus macaque rhadinovirus, *M. nemestrina* RV2, *M. fascicularis* RV2, and Japanese macaque rhadinovirus (*2*,*7*). Complete genome sequences of the RV1 lineage viruses KSHV and RFHV of *M.* *nemestrina* macaques (RFHVMn), as well as of the RV2 lineage viruses rhesus macaque rhadinovirus, Japanese macaque rhadinovirus, and *M. nemestrina* RV2, have been generated from cultured viruses or directly from clinical material by conventional or high-throughput sequencing (*7*–*11*).

Apart from KSHV, all fully sequenced Old World primate rhadinoviruses have been found in primates of the genus *Macaca*, subfamily Cercopithecinae (*7*–*9*,*12*–*14*). We describe a novel rhadinovirus of the Old World primate genus *Colobus* (*14*), subfamily Colobinae, which was detected in a mantled guereza (*Colobus guereza kikuyensis*) that died of primary effusion lymphoma. The virus belongs to the RV1 lineage, together with KSHV and RFHVMn. 

## The Study

**Figure 1:**
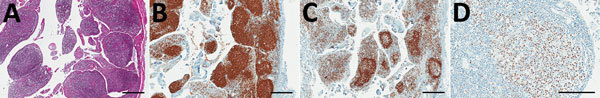
Identification of primary effusion lymphoma and immunohistochemical staining of primary effusion lymphoma cells with Kaposi’s sarcoma–associated herpesvirus latent nuclear-associated antigen (LANA)–specific antibody. A) Diffuse infiltration of the pleura pulmonalis and pleural space with pleomorphic round cells resembling primary effusion lymphoma. Hematoxylin and eosin stain; scale bar indicates 400 μm. B) The neoplastic cells are lymphocytic cells of B cell origin. CD20 immunohistochemistry; scale bar indicates 400 μm. C) Numerous neoplastic cells express the proliferation marker Ki67. Ki67 immunohistochemistry; scale bar indicates 400 μm. D) Typical nuclear expression of a protein related to Kaposi’s sarcoma–associated herpesvirus LANA in neoplastic cells. LANA immunohistochemistry; scale bar indicates 200 μm.

In 2014, a 3-year-old male mantled guereza at a zoo in Germany died suddenly after developing severe anemia (<5 g/dL hemoglobin), subcutaneous edema, and leukocytosis. A necropsy conducted at the German Primate Centre, Göttingen, Germany, led to a diagnosis of primary effusion lymphoma. Large numbers of abnormal leukocytes were found in the vascular system of several organs. The pleura pulmonalis and the pleural space were severely infiltrated with pleomorphic round cells (Figure 1, panel A) identified as CD20-positive B lymphocytes (Figure 1, panel B) with high expression of the proliferation marker Ki67 (Figure 1, panel C). Many neoplastic cells also showed typical nuclear staining with antibodies against the KSHV latent nuclear-associated antigen, suggesting infection with a related herpesvirus (Figure 1, panel D). We detected viral genomes in several organs by using PCR with a panherpesvirus primer set, a primer set specific for the virus detected in this study (colobine gammaherpesvirus 1 [CbGHV1]), or both, for the viral DNA polymerase gene. Sanger sequencing of the panherpes PCR products followed by BLAST (https://blast.ncbi.nlm.nih.gov) analysis revealed the best match to be RFHVMn. Using a commercial microarray (Simian Panel E Kit; Intuitive Biosciences, http://intuitivebio.com), we detected antibodies to lymphocryptovirus but not to simian immunodeficiency virus, simian retrovirus, herpes B virus, simian T-cell leukemia virus, measles virus, rhesus macaque rhadinovirus, human cytomegalovirus, or simian foamy virus (data not shown).

DNA extracted from a spleen necropsy specimen was sequenced by using an Illumina MiSeq (https://www.illumina.com). The 22,978,561 trimmed reads were depleted of host sequences by screening against the human genome sequence. The remaining 3,082,106 reads were assembled de novo into contigs, the largest of which was 126,024 bp. Assemblies of the initial trimmed reads with this sequence, followed by manual extension and incorporation of smaller contigs, resulted in a final, complete viral sequence of 133,999 bp. This essentially circular sequence consists of a unique region (U; 132,514 bp; 52% G+C) followed by a copy of a terminal repeat unit (TR; 758 bp; 84% G+C) and then by a partial copy of TR (727 bp). A total of 84,532 (0.4%) of the initial trimmed reads aligned with this sequence at an average depth of 170 reads per nucleotide. Inspection of the read alignment indicated that most genomes (85%) lack a 7,045-bp region toward the right end of U. In addition, a telomere-like tandem repeat was noted near the left end of U. A similar feature is present in the genome of RFHVMn but not KSHV.

**Figure 2:**
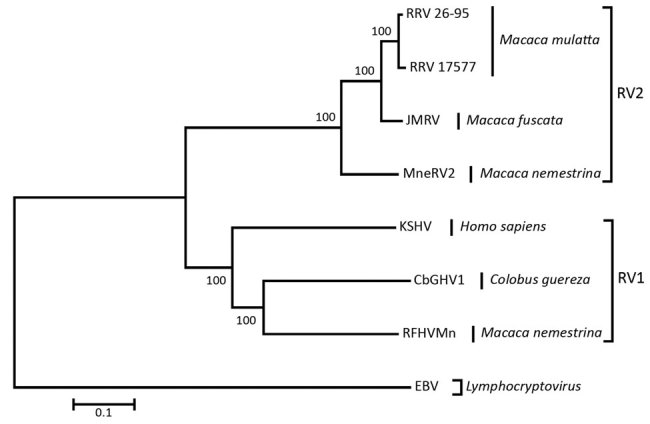
Nucleotide sequence–based phylogenetic analysis of the genomes of CbGHV1 and other gammaherpesviruses. The genus *Lymphocryptovirus* is represented by Epstein-Barr virus as outgroup, and the genus *Rhadinovirus* is represented by the RV1 and RV2 lineages, with host species indicated. Sequences are based on the complete U region, bootstrap values are shown as percentages, and the scale bar represents nucleotide substitutions per site. CbGHV1, colobine gammaherpesvirus 1 (KHSV-like virus isolated from a mantled guereza); EBV, Epstein-Barr virus; JMRV, Japanese macaque rhadinovirus; KSHV, Kaposi’s sarcoma–associated herpesvirus; MneRV2, *Macaca nemestrina*; RV2; RFHVMn, retroperitoneal fibromatosis–associated herpesviruses of *Macaca nemestrina* macaques; RRV, rhesus macaque RV; RV, *Rhadinovirus*.

The viral genome sequence is 51% identical to that of KSHV (137,969 bp) and 59% identical to that of RFHVMn (127,320 bp). Phylogenetic analysis of these 3 sequences with those of viruses in the RV2 lineage, using Epstein-Barr virus (a lymphocryptovirus) as the outgroup, confirmed that the novel virus clusters in the RV1 lineage with RFHVMn and KSHV (Figure 2). The novel virus was thus distinguished from other rhadinoviruses, and we named it colobine gammaherpesvirus 1 (CbGHV1).

We named the 78 protein-coding genes annotated in the CbGHV1 genome according to the KSHV nomenclature (Appendix Figure 1). All are located in U and have orthologs in both KSHV and RFHVMn (Appendix Table). Reanalysis of genome sequences confirmed that RFHVMn contains 82 genes, whereas KSHV contains 86 genes. Several genes first described in KSHV lack orthologs in CbGHV1 (K2, K4.2, K5, K6, K7, and K12) and RFHVMn (K5, K6, and K12). In addition, CbGHV1 lacks open reading frame (ORF) 11 (as does RFHVMn) and ORF49, and ORF2 is truncated. In comparison with RFHVMn, CbGHV1 lacks K2, K4.2, K7, and ORF49. The deletion present in most CbGHV1 genomes affects part of ORF68, all of ORF69, and part of ORF71. Values for percentage amino acid sequence identity between CbGHV1 genes and their counterparts in KSHV and RFHVMn are listed in the Appendix Table. An alignment of the KSHV, RFHVMn, and CbGHV1 latent nuclear-associated antigen (ORF73) sequences showed that all 3 contain the typical extended internal repeat region (Appendix Figure 2).

## Conclusions

We identified and sequenced the complete genome of a novel KSHV-like virus (CbGHV1) from a mantled guereza. The animal died of primary effusion lymphoma, which we assume was caused by CbGHV1. CbGHV1 and its close relatives KSHV and RFHVMn cluster in the RV1 lineage. The presence of a telomere-like tandem repeat near the left end of U in the CbGHV1 and RFHVMn genomes suggests that an ancestral virus may have been integrated into the host genome, and its persistence suggests that these viruses may retain the ability to integrate.

The CbGHV1 genome contains all genes that are conserved in all members of the family *Herpesviridae*. Orthologs of 8 KSHV genes (ORF11, K2, K4.2, K5, K6, K7, ORF49, and K12) are absent from CbGHV1. In KSHV, some of these genes, such as K2 and K12, encode proteins (vIL6 and kaposin, respectively) that have been linked to viral pathogenesis. Their absence from CbGHV1 suggests that they may not be needed for the development of primary effusion lymphoma. Because ORF10 and ORF11 are related and may have arisen by duplication from an ancestral deoxyuridine triphosphatase gene (*15*), their functions may overlap. ORF49 is a cofactor of the KSHV lytic cycle activator Rta (ORF50) and may not be required for the function of CbGHV1 Rta. It is likely that the viral subpopulation lacking all or part of ORF68, ORF69, and ORF71, which encode essential proteins involved in packaging of viral DNA into capsids, egress of capsids from the nucleus, and inhibition of apoptosis, represents a replication-defective deletion mutant generated in the animal investigated. Of note, CbGHV1 has also been identified by PCR in an older female sibling guereza with Kaposi sarcoma–like disease (*16*). The latest offspring of this female guereza were also positive for CbGHV1 but did not show clinical signs. Discovery of CbGHV1 in multiple animals and the determination of its genome sequence may inform future studies of the pathogenesis of primary effusion lymphoma and Kaposi sarcoma, including how KSHV causes primary effusion lymphoma in humans.

Appendix Additional methods and results for study of novel virus related to Kaposi’s sarcoma–associated herpesvirus from colobus monkey.
