# Supplementary material for: Novel Virus Related to Kaposi’s Sarcoma–Associated Herpesvirus from Colobus Monkey
Source: Emerg Infect Dis. 2019 Aug;25(8):1548–51. doi: 10.3201/eid2508.181802 (PMC6649351; doi:10.3201/eid2508.181802)
Supplement: Appendix — Additional methods and results for study of novel virus related to Kaposi’s sarcoma–associated herpesvirus from colobus monkey. [file 18-1802-Techapp-s1.pdf]

Article DOI: https://doi.org/10.3201/eid2508.181802

Novel Virus Related to Kaposi's Sarcoma–Associated Herpesvirus from Colobus Monkey

Appendix

Materials and Methods

Animal and clinical samples

A three-year-old male mantled guereza (*Colobus guereza kikuyensis*) that had been born and housed in Dresden Zoo died suddenly. A necropsy, including histopathologic, immunohistologic and other investigations of several organs, was carried out at the German Primate Center.

Histopathologic examination

Necropsy specimens were prepared as formalin-fixed, paraffin-embedded sections and analysed by hematoxylin and eosin staining. Immunohistochemistry was performed on sections using primary antibodies against human Ki67 (mouse monoclonal antibody, clone MIB-1, DakoCytomation, Hamburg, Germany, 1:50), CD20 (mouse monoclonal anti-human, clone L26, DakoCytomation, 1:300), CD3 (rabbit polyclonal antibody, DakoCytomation, 1:50) and KSHV LANA (rat monoclonal antibody, clone LN35, Abcam, UK, 1:10 recognizes the LANA EEPEPE epitope), respectively, and the streptavidin-biotin-complex method (DAB Map kit, Roche Diagnostics, Germany) in an automated immunostaining system (Discovery XT, Roche).

DNA extraction and partial sequencing

Total DNA was isolated from tissue samples (First-DNA All-Tissue kit, GEN-IAL, Germany). PCR was performed on DNA from lung and spleen samples using the published pan-herpes PCR primer sets DFA, ILK and KG1 (*1*). PCR products extracted from agarose gels were analysed by Sanger sequencing.

Quantitative CbGHV1 specific PCR

Quantitative PCR was conducted in triplicate and repeated three times using 10 ng extracted DNA in 3 mM $MgCl_2$, 0.4 mM deoxynucleoside triphosphates, 0.266 μM probe, 0.6 μM (each) sense and antisense primers, 1 x PCR buffer (Qiagen, Germany) and 0.25 μl HotStarTaq DNA polymerase (Qiagen). The thermal profile was 95 °C for 15 min followed by 45 cycles consisting of 15 s at 95 °C and 1 min at 60 °C on a Rotor-Gene Q (Qiagen). Primers and probe were based on the CbGHV1 DNA polymerase catalytic subunit (ORF9) gene sequence (5´-CCGAGACAGTAACCCTCCAA-3´, 5´-TTAGCAGGCAGGCTAAGTGT-3´, and 5´FAM-TGGCTTCCACGAAGACCTGTGACT-3´BHQ-1).

Genome sequencing

Sequencing libraries were prepared from DNA extracted from a spleen sample by using a KAPA library preparation kit (KAPA Biosystems, USA). Fragments were generated by sonication, end-repaired, A-tailed, ligated to the NEBnext Illumina adaptor (New England BioLabs, USA) and amplified by PCR using a KAPA HiFi real-time library amplification kit on an ABI 7500 real-time cycler (Applied Biosystems, USA). After quality control using a Qubit 2.0 fluorometer (Invitrogen, USA) and a Bioanalyzer (Agilent Technologies, USA), sequencing was performed on an Illumina MiSeq using a v. 3 reagent kit (Illumina), generating a dataset of 300 nucleotide paired-end reads.

Host sequences were removed by mapping the reads to the UCSC hg19 human reference genome. The remaining unmapped reads were quality-filtered (FastQC v. 0.11.5), and adapter sequences were removed (Trim Galore v. 0.4 (http://www.bioinformatics.babraham.ac.uk/projects/trim_galore). Consensus sequences were derived *de novo* (SPAdes v. 3.10.1 (*2*)), and read assemblies were generated and checked manually (CLC genomics workbench v. 9 (Qiagen)) or generated using Bowtie 2 v. 2.3.1 (*3*) and visualized (Tablet v. 1.17.08.17 (*4*)). The viral genome sequence was annotated by comparison with the KSHV and RFHVMn genome sequences (Geneious v. 11.1.3 (*5*). Using standard bioinformatics tools, searches were also conducted for potentially novel genes that had been missed in previous analyses of the KSHV and RFHVMn sequences.

Sequence alignments and phylogenetic analyses

Nucleotide sequence alignments of the genome sequences of CbGHV1, KSHV (AF148805), RFHVMn (KF703446), EBV (NC_007605), RRV strain 26-95 (AF210726), RRV strain 17577 (AY528864), JMRV (AY528864) and MneRV2 (KP265674) were constructed using MAFFT v. 7 (*6*). Phylogenetic analyses were carried out using MEGA v. 7 (*7*), employing the neighbor-joining method with 1000 bootstrap replicates. Amino acid sequence alignments were constructed for individual genes using Bioedit v. 7.2.0 or Geneious v. 11.1.3 (*5*) to calculate percentage identity.

Genome sequence accession number

The CbGHV1 genome sequence was deposited in NCBI GenBank (accession number MH932584).

References

1. Chmielewicz B, Goltz M, Lahrmann KH, Ehlers B. Approaching virus safety in xenotransplantation: a search for unrecognized herpesviruses in pigs. Xenotransplantation. 2003;10:349–56. PubMed http://dx.doi.org/10.1034/j.1399-3089.2003.02074.x

2. Bankevich A, Nurk S, Antipov D, Gurevich AA, Dvorkin M, Kulikov AS, et al. SPAdes: a new genome assembly algorithm and its applications to single-cell sequencing. J Comput Biol. 2012:19:455-77.

3. Langmead B, Salzberg SL. Fast gapped-read alignment with Bowtie 2. Nat Methods. 2012;9:357–9. PubMed http://dx.doi.org/10.1038/nmeth.1923

4. Milne I, Stephen G, Bayer M, Cock PJ, Pritchard L, Cardle L, et al. Using Tablet for visual exploration of second-generation sequencing data. Brief Bioinform. 2013;14:193–202. PubMed http://dx.doi.org/10.1093/bib/bbs012

5. Kearse M, Moir R, Wilson A, Stones-Havas S, Cheung M, Sturrock S, et al. Geneious Basic: an integrated and extendable desktop software platform for the organization and analysis of sequence data. Bioinformatics. 2012;28:1647–9. PubMed http://dx.doi.org/10.1093/bioinformatics/bts199

6. Katoh K, Misawa K, Kuma K, Miyata T. MAFFT: a novel method for rapid multiple sequence alignment based on fast Fourier transform. Nucleic Acids Res. 2002;30:3059–66. PubMed http://dx.doi.org/10.1093/nar/gkf436

7. Kumar S, Stecher G, Tamura K. MEGA7: Molecular Evolutionary Genetics Analysis version 7.0 for bigger datasets. Mol Biol Evol. 2016;33:1870–4. PubMed http://dx.doi.org/10.1093/molbev/msw054

Appendix Table. Functions of CbGHV1 genes and amino acid sequence comparisons with orthologous genes in KSHV, RFHVMn, and RRV*

Gene	Protein product	Identity to RFHVMn (%)	Identity to KSHV (%)	Identity to RRV (%)
K1	Membrane glycoprotein K1	36.3	13.2	18.8
ORF4	Complement control protein	44.4	37.7	25.3
ORF6	Single-stranded DNA-binding protein	78.6	72.9	61.2
ORF7	DNA packaging terminase subunit 2	67.4	62.9	51.4
ORF8	Envelope glycoprotein gB	71.7	66.7	60.1
ORF9	DNA polymerase catalytic subunit	81.0	73.9	66.7
ORF10	Protein G10	56.7	40.8	32.6
ORF2	Dihydrofolate reductase	41.8	37.5	38.3
K3	E3 ubiquitin ligase MIR1	44.4	31.1	19.4
ORF70	Thymidylate synthase	69.8	60.7	62.6
K4	CC chemokine vCCLl2	57.4	54.3	17.0
K4.1	CC chemokine vCCL3	58.3	40.3	19.0
ORF16	Apoptosis regulator G16	53.9	46.3	37.0
ORF17	Capsid maturational protease	60.3	49.8	41.5
ORF17.5	Capsid scaffold protein	49.4	33.8	25.6
ORF18	Protein UL79	71.9	65.4	56.8
ORF19	DNA packaging tegument protein UL25	71.5	60.7	49.6
ORF20	Nuclear protein UL24	61.0	49.6	37.2
ORF21	Thymidine kinase	60.6	49.0	36.0
ORF22	Envelope glycoprotein gH	61.1	43.6	36.0
ORF23	Protein UL88	43.9	38.4	24.6
ORF24	Virion protein UL87	70.2	66.5	53.7
ORF25	Major capsid protein	88.9	82.4	73.5
ORF26	Capsid triplex subunit 2	82.3	77.4	59.0
ORF27	Envelope glycoprotein 48	51.4	42.2	25.3
ORF28	Envelope glycoprotein 150	47.3	40.8	21.5
ORF29	DNA packaging terminase subunit 1	78.1	73.7	57.3
ORF30	Protein UL91	62.3	58.8	33.3
ORF31	Protein UL92	75.9	70.1	47.3
ORF32	DNA packaging tegument protein UL17	55.6	44.1	37.4
ORF33	Tegument protein UL16	75.5	61.2	42.9
ORF34	Protein UL95	69.6	61.8	47.4
ORF35	Tegument protein UL14	65.3	53.3	30.9
ORF36	Tegument serine/threonine protein kinase	72.1	61.7	43.1
ORF37	Deoxyribonuclease	82.9	70.6	65.7
ORF38	Myristylated tegument protein	59.4	53.1	43.9
ORF39	Envelope glycoprotein gM	75.6	63.0	58.9
ORF40	Helicase-primase subunit	51.6	43.3	30.0
ORF42	Tegument protein UL7	70.8	60.6	46.0
ORF43	Capsid portal protein	80.1	74.7	60.3
ORF44	Helicase-primase Helicase subunit	83.5	76.1	67.0
ORF45	Tegument protein G45	52.1	35.1	22.6
ORF46	Uracil-DNA glycosylase	77.6	67.1	54.2
ORF47	Envelope glycoprotein gL	57.3	39.6	31.9
ORF48	Tegument protein G48	49.0	32.3	29.2
ORF50	Protein Rta	54.1	49.7	37.5
K8	Protein Zta	54.4	29.7	12.9
K8.1	Glycoprotein gp350	28.9	21.0	12.4
ORF52	Virion protein G52	66.9	53.0	42.6
ORF53	Envelope glycoprotein gN	56.7	47.3	45.9
ORF54	Deoxyuridine triphosphatase	67.6	48.1	37.8
ORF55	Tegument protein UL51	69.4	64.8	51.9
ORF56	Helicase-primase primase subunit	66.9	58.6	50.2
ORF57	Mutifunctional expression regulator	64.5	55.8	40.4
K9	Interferon regulatory factor 1	53.6	36.9	17.7
K10	Interferon regulatory factor 4	38.6	24.8	NA
K10.5	Interferon regulatory factor 3	34.1	24.4	NA
K11	Interferon regulatory factor 2	35.8	26.1	NA
ORF58	Envelope protein UL43	62.9	49.6	36.7

Gene	Protein product	Identity to RFHVMn (%)	Identity to KSHV (%)	Identity to RRV (%)
ORF59	DNA polymerase processivity subunit	65.8	57.8	47.1
ORF60	Ribonucleotide reductase subunit 2	84.9	75.7	70.5
ORF61	Ribonucleotide reductase subunit 1	80.1	66.9	61.3
ORF62	Capsid triplex subunit 1	76.2	64.4	51.8
ORF63	Tegument protein UL37	57.3	47.3	37.4
ORF64	Large tegument protein	56.6	46.8	36.2
ORF65	Small capsid protein	55.1	45.2	35.7
ORF66	Protein UL49	62.6	57.3	42.7
ORF67	Nuclear egress membrane protein	75.8	59.4	56.6
ORF67A	DNA packaging protein UL33	72.6	60.7	57.0
ORF68	DNA packaging protein UL32	60.0	59.1	49.2
ORF69	Nuclear egress lamina protein	78.7	66.2	60.4
ORF71	Apoptosis regulator FLIP	50.8	45.5	35.6
ORF72	Cyclin	46.3	45.6	37.5
ORF73	Nuclear antigen LANA-1	46.3	26.3	14.6
K14	Glycoprotein CD200	57.1	37.5	31.2
ORF74	Membrane protein G74	65.4	56.2	42.4
ORF75	Tegument protein G75	75.6	60.5	42.1
K15	Membrane protein K15	47.1	20.5	11.6

*CbGHV1, KHSV-like virus isolated from a mantled guereza; KSHV, Kaposi sarcoma–associated herpesvirus; LANA, latent nuclear-associated antigen; NA, not applicable; ORF, open reading frame; RFHVMn, retroperitoneal fibromatosis–associated herpesviruses identified in *Macaca nemestrina* macaques; RRV, rhesus macaque rhadinovirus

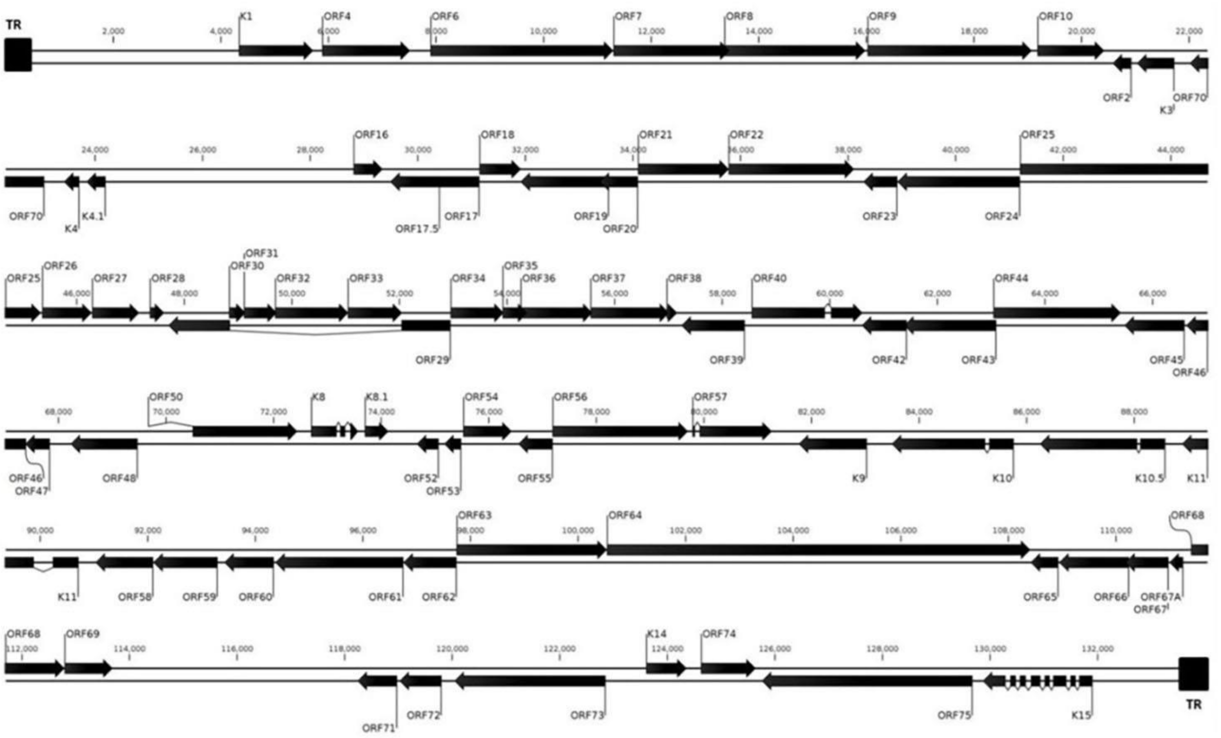

Appendix Figure 1. CbGHV1 genome map. The genome is depicted as U flanked at each end by a single copy of TR, although variable numbers of TR are likely present at each end. Protein-coding regions are represented as black arrows. The scale is in base pairs (bp).

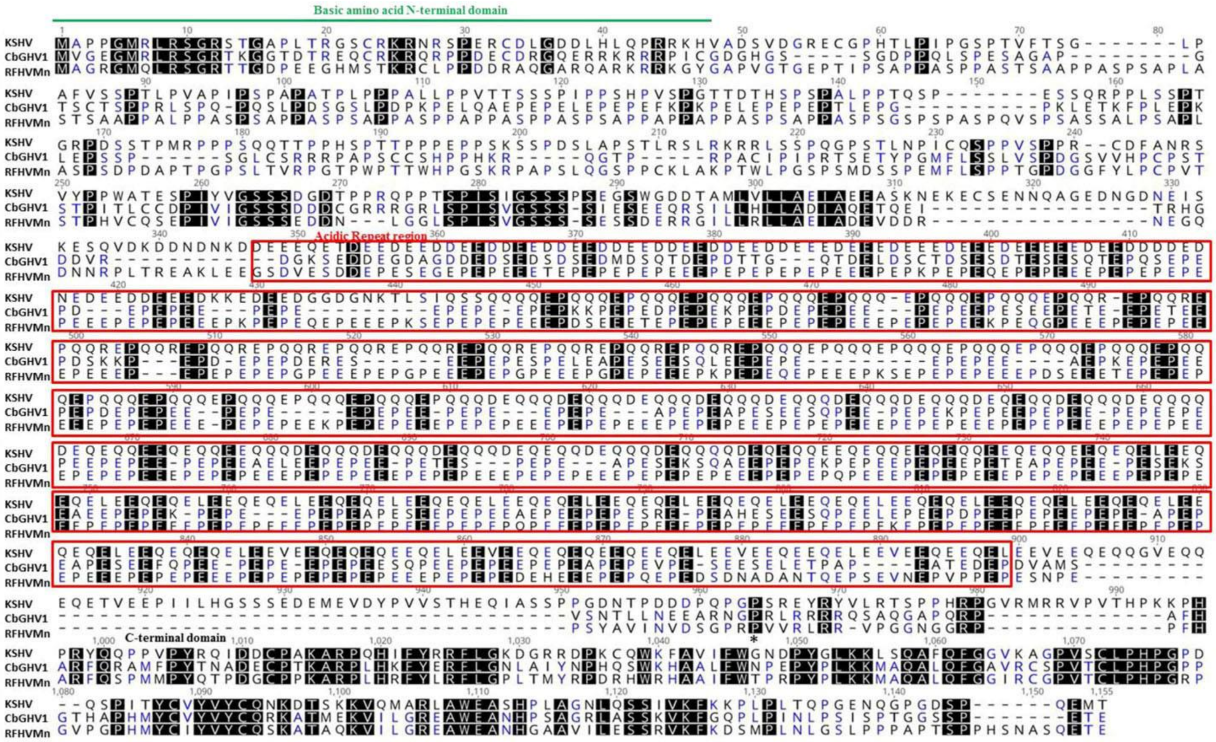

Appendix Figure 2. Amino acid sequence alignment of LANA (ORF73) for members of the RV1 lineage. Identical residues in three sequences are highlighted, and identical residues in two sequences are in blue font. The N-terminal domain is indicated in green font and the acidic repeat region in red font, and the C-terminal domain starts with an asterisk at residue 964.
